# Pre-job loss grief reactions and work attachment among sick-listed employees: Introduction of the imminent Job Loss Scale

**DOI:** 10.1186/s40359-024-01626-8

**Published:** 2024-03-02

**Authors:** Janske H. W. van Eersel, Inge L. Hulshof, Miriam I. Wickham, Geert E. Smid, Paul A. Boelen

**Affiliations:** 1 Department Human Resource Studies, Tilburg University, Tilburg, Postbus 90153, 5000 The Netherlands; 2 Department of Clinical Psychology, Utrecht University, Utrecht, The Netherlands; 3 Department of Work and Organizational Psychology, Open University, Heerlen, The Netherlands; 4 ZINZIZ, Utrecht, The Netherlands; 5 Department of Social, Health and Organisational Psychology, Utrecht University, Utrecht, The Netherlands; 6 University of Humanistic Studies, Utrecht, The Netherlands; 7 ARQ National Psychotrauma Centre, Diemen, The Netherlands

**Keywords:** Imminent job loss, Organizational commitment, Pre-loss grief, Re-integration, Sick-listed employees, Work attachment

## Abstract

**Background:**

With this study, we aimed to explore the emotional experiences of sick-listed employees facing imminent job loss, as this emotional distress may hinder successful job search outcomes. The study had two objectives: (1) to develop and validate the Imminent Job Loss Scale (IJLS) for assessing pre-job loss grief reactions and (2) to examine its relationship to work attachment.

**Methods:**

Development of the 9-item IJLS was carried out using feedback from an expert panel, consisting of five academic experts in grief and labour, five re-integration specialists, and five sick-listed employees facing imminent job loss. The psychometric properties of the IJLS were evaluated, and its association with work attachment was examined using data from 200 sick-listed employees facing imminent job loss.

**Results:**

The IJLS demonstrated strong internal consistency and temporal stability, distinctiveness from depression and anxiety symptoms, and solid convergent validity. Work-centrality and organizational commitment were positively related to pre-job loss grief reactions, while work engagement and calling showed no significant associations.

**Conclusion:**

This study provides valuable insights into pre-job loss grief reactions and shows the potential utility of the IJLS for screening and monitoring purposes. Understanding pre-job loss grief reactions can improve the re-integration and job prospects of sick-listed employees. In future research, explorations of these dynamics should continue to provide better support to sick-listed employees during this challenging period.

**Supplementary Information:**

The online version contains supplementary material available at 10.1186/s40359-024-01626-8.

## Introduction

In the Netherlands, the sickness absence rate among employees is 4.8% [1]. Employers are responsible for wage contribution and re-integration for their sick-listed employees over a period of 104 weeks [2]. Typically, after one year of absence, sick-listed employees are required to explore external job opportunities alongside attempting to return to work and searching for job opportunities within their current company. Balancing the intricate interplay of uncertainties associated with imminent job loss, the internal job search, health problems, recovery prospects, future opportunities, and the imperative of pursuing external job options may lead to severe emotional distress [3]. This type of distress can hinder the quality of their job search [4], resulting in only 7% securing external employment, 26% finding suitable jobs within their current organization, and 67% experiencing job loss after 104 weeks [2]. With this study, we aimed to address the emotional experiences of sick-listed employees facing imminent job loss, by exploring pre-job loss grief reactions.

Job loss can elicit a range of negative emotional responses, including symptoms of depression, anxiety [5], and job loss-related grief [6]. Extensive research consistently differentiates the constructs of depression, anxiety, and grief that manifest after involuntary job loss [6–8]. Furthermore, study results have revealed that factors like duration of employment and the cause of job loss (e.g., health issues, organizational changes, or labour disputes) are not correlated with the intensity of job loss-related grief symptoms [9–11]. Building on the work of Greenhalgh and Rosenblatt [12], who established a connection between job insecurity and grief, it seems conceivable that sick-listed employees might, to some extent, experience pre-job loss grief reactions due to their imminent job loss, irrespective of the severity of the health problems they experience.

Although grieving processes differ between bereavement and non-bereavement losses, there are also some similarities in grief reactions associated with job loss [13], natural disaster [14], climate change [15], and romantic break-ups [16]. Studies among family caregivers of terminally ill patients have shown that individuals can also exhibit anticipatory or pre-loss grief reactions [17]. These reactions are predictive of symptoms of prolonged grief after their loved one’s death [18]. It is conceivable that sick-listed employees facing imminent job loss might also experience pre-job loss grief reactions [12]. Mirroring the conceptualization of job loss-related grief [19], pre-job loss grief reactions are likely to involve difficulties in accepting the changed reality, yearning for the state prior to the imminent job loss, preoccupation with the impending job loss, feelings of anger and loneliness, identity disruption and problems with finding purpose. In this study, we examined pre-job loss grief reactions by developing and validating an instrument to assess such reactions. Understanding this phenomenon could enhance re-integration and job prospects for sick-listed employees, as mental health problems may hinder successful re-employment [20].

Several psychological mechanisms may be associated with increased pre-job loss grief. Building upon the concept of job loss-related grief [21], individuals with a stronger *work attachment* may be more prone to experience pre-job loss grief reactions. An important aspect to consider is the latent nature characterizing the quantification of work attachment, necessitating the integration of foundational elements to assess it. For instance, *work-centrality* (i.e., the impact of work on one’s identity) is associated with higher levels of identity disruption following job loss [13]. Another indicator of work attachment is *work engagement*, reflecting an individual's positive and fulfilling involvement in their work. Highly engaged employees invest significant effort and personal resources into their work. However, disruption in the equilibrium between giving and taking can lead to emotional distress [22]. Additionally, employees who perceive their work as a *calling* (i.e., meaningful and prosocial orientated work), yet are unable to live out their calling, may experience distress [23]. Furthermore, concerns about job loss can result in a stronger *organizational commitment* (i.e., one's psychological attachment to the organization) [24], which is often observed among sick-listed employees [3]. Consequently, the present study employed four fundamental components – namely work-centrality, work engagement, calling, and organization commitment – to assess the intricate concept of work attachment. The rationale for selecting these components as indicators lies in the anticipation that sick-listed employees with a stronger work attachment might be more susceptible to experiencing pre-job loss grief reactions throughout the 104-week sick leave period.

### Present study

**Fig. 1:**
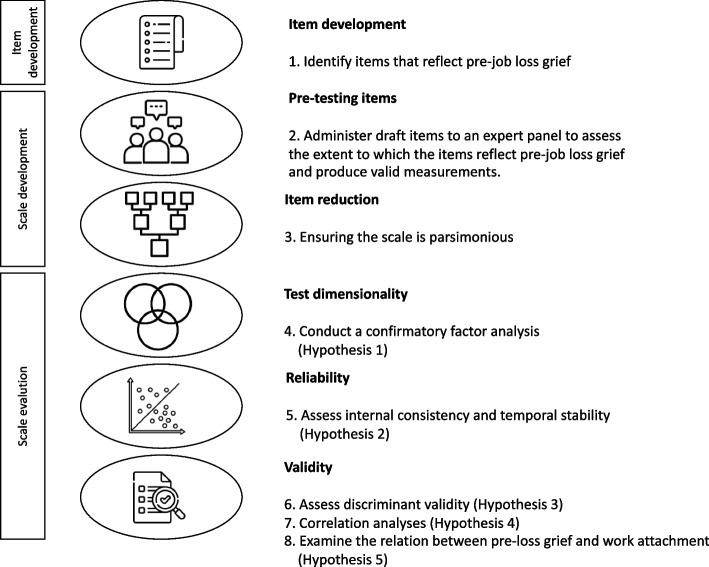
Overview development and validation of the Imminent Job Loss Scale

For the development and validation of the Imminent Job Loss Scale (IJLS), we adhered the procedural framework delineated by Boateng and colleagues [25], encompassing three phases: 1) item development, 2) scale development, and 3) scale evaluation. See Fig. 1 for an overview.

There appear to be commonalities in the grieving process following various losses [13–16], including reduction of physical, psychological, and symbolic resources, the search for meaning, hope and agency, and the development of new identity incorporating the loss [26]. Pre-job loss grief is a relatively new concept; hence, we chose to utilize the Job Loss Grief Scale [8] as a foundational basis for item development of the IJLS (Phase 1), since this scale measures alleged markers of grief and has adequate psychometric properties.

During Phase 2, scale development, we obtained input from an expert panel, who evaluated all items. The assessment of the expert panel was employed to reduce items and formulate a parsimonious scale.

Regarding scale evaluation (Phase 3), we examined various psychometric properties of the IJLS and its relation to work attachment, using data from 200 sick-listed employees facing imminent job loss. In terms of dimensionality, based on prior research on grief instruments [8, 27], we anticipated that the IJLS would demonstrate a similar unidimensional structure (Hypothesis 1). As for reliability, we expected the scale to exhibit good internal consistency and short-tem temporal stability (Hypothesis 2). Concerning validity, prior research has shown that symptoms of grief, depression, and anxiety manifest in distinct clusters [7, 8, 28]. Hence, we assumed that items related to pre-job loss grief, depression, and anxiety would fall into three different symptom clusters (Hypothesis 3). Previous studies indicated a relation between grief on the one hand and depression, anxiety, coping styles, quality of life, perceived health, optimism [6, 13, 19], and intolerance of uncertainty on the other hand [29]. Therefore, we expected that higher levels of pre-job loss grief would be positively associated with depression, anxiety, denial, intolerance of uncertainty, and negatively associated with quality of life, perceived health, acceptance, and optimism (Hypothesis 4). Finally, we hypothesized a positive association between pre-job loss grief reactions and work attachment manifestations, including work centrality, work engagement, calling, and organizational commitment [13, 22–24]. We expected these associations to remain significant after controlling for elapsed time since starting the external job search, reported financial strain, and quality of employee-employer communication [3] (Hypothesis 5).

## Method

### Procedure and participants

This study is part of a larger project, titled ‘Aligning willingness and ability, to set the right course’, that aims to study and improve re-integration of sick-listed employees.

For Phase 2, scale development, a panel of five experts specialized in grief and labour, five professionals specialized in re-integration, and five sick-listed employees facing imminent job loss were recruited from the authors’ social network. The panel participated in the development of the IJLS by assessing items related to pre-job loss grief. Participants provided informed consent.

For Phase 3, scale evaluation, sick-listed employees legally required to seek for external job opportunities were recruited between November 2022 and January 2023. Participants were recruited through social media channels (e.g., LinkedIn) and re-integration agencies that informed their clients about the research. Participants signed an informed consent form (*N* = 232), after which 86% completed the survey. Out of the participants, 194 completed the survey online (Qualtrics) in approximately 15 min, while a subset of six preferred to the complete the survey during a 45-min phone interview with the first author. To assess the temporal stability, 167 participants (84%) who completed the survey filled out the IJLS twice (T_2_), with a test–retest interval ranging from 4 to 18 (*M* = 7.7; *SD* = 2.7) days. Ethical approval was obtained from the Ethics Review Board of the Faculty of Social and Behavioural Sciences at Utrecht University (Phase 2, FETC 22–0430; Phase 3, FETC 22–0504).

### Measures

Data on socio-demographic variables (e.g., age), work-related factors (e.g., duration of employment), and health issues (e.g., perceived limitations) were collected (see Table 1).

**Table 1:** Characteristics of the participants

**Socio-demographics**	*N (%)*	*M*	*SD*
Gender			
Male	31 (16)		
Female	169 (84)		
Age in years		49.0	9.9
Education			
Elementary school	1 (1)		
Vocational secondary education	8 (4)		
Secondary education	45 (22)		
Academic education	146 (73)		
**Work characteristics**			
Weekly work hours		29.9	7.1
Years of employment		11.7	10.1
Start external job search			
< 1 month ago	44 (22)		
1–3 months ago	33 (16)		
3–6 months ago	37(19)		
6–9 months ago	19 (10)		
> 9 months ago	45 (22)		
missing	22 (11)		
Work status			
Working internally	78 (39)		
Working externally	24 (12)		
Not working	98 (49)		
**Scale characteristics**			
Pre-job loss grief (IJLS)		28.7	7.7
Depression (HADS)		16.1	4.3
Anxiety (HADS)		17.2	4.3
Denial (Brief COPE)		4.5	1.7
Intolerance of uncertainty (IUS-12)		38.0	7.9
Quality of life (ICECAP-A)		12.8	2.3
Perceived health (EQ-5D-NL)		49.4	16.4
Acceptance (Brief COPE)		4.7	1.4
Optimism (LOT-R)		19.8	4.1
Financial strain		1.7	0.7

For Phase 1, item development, the IJLS was based on the 33-item Job Loss Grief Scale, which taps into job loss-related complicated grief reactions [8]. For Phase 2, an expert panel rated the items on a 5-point Likert scale (1 = *poor* to 5 = *excellent*) for comprehensibility (‘To what extent is this item comprehensible?’), content validity (‘To what extent does this item represent pre-job loss grief’) and face validity (‘What rating would you give this item?’). For Phase 3, participants rated to extent to which they experienced pre-job loss grief reactions in the preceding four weeks on a 5-point scale (1 = *never* to 5 = *always*). The IJLS items are presented in Table 2, and the Dutch version is provided in the Appendix.

**Table 2:** Confirmatory factor loadings

	CFA		CFA	
	**Pre-job loss grief**	**Pre-job loss grief**	**Depression**	**Anxiety**
*Items of the imminent job loss scale*				
1. I longed strongly for how my life was before theimpending loss of my job	.59	.58		
2. I constantly thought about the impending loss of my job	.82	.81		
3. I was angry about the impending loss of my job	.66	.69		
4. I could hardly believe that I am at risk of losing my job	.79	.80		
5. My future seemed meaningless because of the impending loss of my job	.78	.80		
6. I felt emotionally numb due to the impending loss of my job	.87	.85		
7. I did everything I could to avoid thinking about the impending loss of my job	.78	.77		
8. I no longer knew who I was because of the impending job loss	.75	.80		
9. I felt lonely because of my impending job loss	.66	.78		
*Items of the HADS – depression scale*				
1. I still enjoy the things I used to enjoy			.72	
2. I can laugh and see the funny side of things			.81	
3. I feel cheerful			.82	
4. I feel as if I am slowed down			.76	
5. I have lost interest in my appearance			.61	
6. I look forward with enjoyment to things			.80	
7. I can enjoy a good book or radio or TV program			.76	
*Items of the HADS – anxiety scale*				
8. I feel tense or 'wound up'				.85
9. I get a sort of frightened feeling as if something awful is about to happen				.80
10. Worrying thoughts go through my mind				.78
11. I can sit at ease and feel relaxed				.82
12. I get a sort of frightened feeling like 'butterflies' in the stomach				.65
13. I feel restless as I have to be on the move				.77
14. I get sudden feelings of panic				.76

Participants completed validated Dutch versions of the instruments unless otherwise indicated. *Anxiety* (Cronbach’s α=0.87) and *depression* (α=0.86) symptoms were measured with the 14-item Hospital Anxiety and Depression Scale (HADS) [30]. Items (.e.g., ‘I feel tense or wound up') were rated on a 4-point scale (e.g., 1 = *not at all* to 4 = *nearly all the time*). Coping strategies were assessed with two subscales, *denial* (α=0.74) and *acceptance* (α=0.75), of the Brief COPE [31]. Items were rated on a 4-point scale (1 = *never or rarely* to 4 = *very frequently*). A sample item is ‘I’ve been learning to live with it.’ The Investigating Choice Experiments Capability – Adult (ICECAP-A; α=0.75) was used to measure *quality of life* [32]. The instrument covers five dimensions of life (stability, attachment, autonomy, achievement and enjoyment), each rated by participants on 4-level response scale (1 = *no capability* to 4 = *full capability*) to denote their current overall quality of life. *Intolerance of uncertainty* (α=0.88) was assessed with the Intolerance of Uncertainty Scale—Short Form (IUS-12) [33]. Items (e.g., ‘I always want to know what the future has in store for me’) were rated on a 5-point scale *(*1 = *Not at all representative* to 5 = *Completely representative). Optimism* (α=0.84) was measured with the 6-item Life Orientation Test-Revised (LOT-R) [34]. Items (e.g., ‘In uncertain times, I usually expect the best’) were rated on a 5-point scale (1 = *strongly disagree* to 5 = *strongly agree*). Perceived health was assessed with one item of the EQ-5D-NL [35]. Participants were asked to rate their perceived health over the last three months on a scale from 0 (*worst possible health they can image*) to 100 (*best possible health they can image*).

*Work centrality* (α=0.61) was assessed with three statements [36]. Items were rated on a 5-point scale (1 = *strongly disagree* to 5 = *strongly agree*). A sample item is ‘The major satisfaction in my life comes from my job’. *Work engagement* (α=0.94) was tapped into with the 3-item subscale ‘dedication’ of the Utrecht Work Engagement Scale (UWES-9) [37]. Items were rated on a 6-point scale (1 = *never* to 6 = *always*). A sample item is ‘I am enthusiastic about my job’. *Organizational commitment* (α=0.91) was assessed with the 3-item affective commitment subscale [38]. Items were rated on a 5-point scale (1 = *not at all* to 5 = *absolutely*). A sample item is ‘I feel emotionally attached to this organization’. *Calling* (α=0.91) was measured using the case description ‘calling’ of the University of Pennsylvania Work-Life questionnaire [39] capturing the extent to which individuals identify their current job as a calling. The case description was translated with the forward and back method. Participants rated their level of identification on 5-point scale (1 = *not at all* to 5 = *very strong*). Quality of employee-employer communication (α=0.91) was assessed with two modified items from the leader-member exchange model [40], namely ‘The communication with my employer is positive and constructive’ and ‘I feel valued by my employer’. Items were rated on a 5-point scale (1 = *strongly disagree* to 5 = *strongly agree*). Financial strain was assessed with a single item ‘How is your financial situation at the end of the month?’ [41] and rated on 3-point scale (1 = *Usually I have money to spare* to 3 = *Usually I have not enough money to make ends meet*).

### Statistical analyses

Analyses were conducted in Mplus (Version 8.7) [42] and SPSS version 29. For Phase 2, the Intraclass Correlation Coefficient (ICC), a Two-Way Random model, was calculate based on all assessments of the expert panel.

For Phase 3, items of IJLS and HADS were marked as categorical. Confirmatory factor analysis (CFA) was used to test Hypothesis 1, evaluating the unidimensional structure of the IJLS. Model fit was evaluated using: χ^2^-value, χ^2^/*df* ratio, Tucker-Lewis index (TLI), root mean square error of approximation (RMSEA), and standardized root mean square residual (SRMR). Lower χ^2^ and χ^2^/*df* indicate better fit [43]. Acceptable fit was defined as TLI > 0.90 and RMSEA and SRMR < 0.08 [44]. Internal consistency of the IJLS was tested with Cronbach’s alpha and temporal consistency was tested with Pearson correlation of T_2_ (Hypothesis 2). Discriminant validity between the IJLS and HADS items was examined through CFA (Hypothesis 3). Correlation analyses were employed to examine Hypothesis 4, which assessed associations between pre-job loss grief reactions and depression, anxiety, denial, quality of life, perceived health, acceptance, and optimism. As a final test of validity, a multiple regression analysis (MRA) was conducted to examine the association between various manifestations of work attachment and pre-job loss grief reactions. Additionally, a hierarchical multiple regression analysis (HMRA) was performed, with pre-job loss grief reactions as dependent variable and various manifestations of work attachment as independent variables, controlling for elapsed time since starting the external job search, perceived financial strain, and quality of employee-employer communication (Hypothesis 5).

## Results

### Item and scale development

In Phase 1, the authors modified all 33 items from the Job Loss Grief Scale to refer to pre-job loss grief. For instance, ‘I think about the loss of my job all the time’, became ‘I constantly thought about the impending loss of my job’.

In Phase 2, the mean scores of the expert panel assessments were calculated, ranging from 3.7 to 5.0 for comprehensibility, 3.4 to 4.9 for content validity, and 3.0 to 4.7 for face validity (see Supplementary File). The ICC for inter-rater reliability was 0.53 (95% CI: 0.45—0.60), which indicates a moderate reliability [45].

Our primary focus was to encompass all essential aspects of pre-job loss grief, including difficulties in accepting the changed reality, yearning, preoccupation with the impending job loss, feelings of anger and loneliness, identity disruption, and problems with finding purpose.

During the item reduction process, it became evident that these aspects were covered by nine items, all scoring 4.6 or higher on the combined mean. In cases where multiple items addressed the same facet of pre-job loss grief, the item with the most favourable score was selected. The aim was to strike a balance between brevity and inclusiveness, ensuring that the instrument remains comprehensive while minimizing participant burden. Consequently, nine items were retained for the IJLS.

#### Characteristics of the expert panel

The five grief and labour experts of the panel comprised of one male and four females, with an average age of 42.4 years (*SD* = 6.7), all holding a PhD, and having an average of 16.4 years (*SD* = 4.8) of relevant work experience. The five re-integration professionals of the panel included two males and three females, with an average age of 58.5 years (*SD* = 5.1), holding an applied university degree, and an average of 14.8 years (*SD* = 7.3) of relevant work experience. The five sick-listed employees of the panel were highly educated women with an average age of 47.5 years (*SD* = 11.5).

### Scale evaluation

#### Participants characteristics

Table 1 presents the participants’ socio-demographics and work characteristics, who participated in Phase 3. The participants experienced limitations in one or more areas due to health issues: physical (*N* = 120), mental (*N* = 145), energetic (*N* = 145), and other (*N* = 19). The IJLS score was analysed in relation to socio-demographics (age, gender, and educational level) and work characteristics (weekly work hours, duration of employment, and work status). All variables showed a non-significant relation, except for age and work status. Higher age was associated with a higher level of pre-job loss grief reactions (*r* = 0.21, *p* = 0.003). Work status was significantly related to pre-job loss grief (*F* (2,197) = 3.60, *p* = 0.029). Participants who worked within their current organization scored significantly lower on pre-job loss grief (*M* = 26.9) than participants who worked externally (*M* = 30.5; *p* = 0.042) or were not working (*M* = 29.6; *p* = 0.019). There was no significant difference (*p* = 0.599) for pre-job loss grief between individuals who worked externally or those who were not working.

#### Dimensionality of the IJLS

Regarding the dimensionality, the one-factor model with all items loading on a single latent factor yielded acceptable model fit, χ^2^ = 148.01, *df* = 27; χ^2^/*df* = 5.48; TLI = 0.94; RMSEA = 0.09; SRMR = 0.05. The high RMSEA value appeared to be due to correlations between three pairs of items (items 3 and 4; items 5 and 9; items 8 and 9). Allowing these pairs to correlate substantially improved the model fit: χ^2^ = 56.58; *df* = 24; χ^2^/*df* = 2.36; TLI = 0.98; RMSEA = 0.08; SRMR = 0.03, which confirmed Hypothesis 1.

#### Reliability of the IJLS

All factor loadings ranged from 0.59 to 0.87 (see Table 2). As for reliability, Hypothesis 2 was supported, with high internal consistency (α = 0.90) and temporal stability between T_1_ and T_2_ (*r* = 0.81).

#### Validity of the IJLS

In relation to validity, the CFA three-factor model with pre-job loss grief, depression, and anxiety symptoms as distinct latent factors, yielded an acceptable model fit, χ^2^ = 424.44, *df* = 227; χ^2^/*df* = 1.87; TLI = 0.95; RMSEA = 0.07; SRMR = 0.06. The latent factors were correlated: pre-job loss grief with depression (*r* = 0.43); pre-job loss grief with anxiety (*r* = 0.51); depression with anxiety (*r* = 0.79). Hence, the discriminant validity passed the Fornell-Larcker criterion [46], confirming Hypothesis 3. Factor loadings are presented in Table 2.

Hypothesis 4 was supported, as pre-job loss grief reactions were positively associated with depression (*r* = 0.37), anxiety, (*r* = 0.44), denial (*r* = 0.51), and intolerance of uncertainty (*r* = 0.43), and negatively related to quality of life (*r* = -0.33), perceived health (*r* = -0.11), acceptance (*r* = -0.37), and optimism (*r* = -0.36).

Regarding Hypothesis 5, the MRA yielded significant results (*R*^*2*^ = 0.20, *F* (4, 195) = 12.29, *p* < 0.001). Work-centrality (β = 0.38; *p* < 0.001) demonstrated a significant association with pre-job loss grief reactions, as opposed to work engagement (β = 0.06; *p* = 0.49), organizational commitment (β = 0.05; *p* = 0.50), and calling (β = 0.05; *p* = 0.54). In the HMRA, elapsed time since starting the external job search, reported financial strain, and quality of employee-employer communication explained 9% of the variance of pre-job loss grief reactions. After adding work attachment variables to the model, significant results emerged (*R*^*2*^ = 0.28, *F* (4, 169) = 12.71, *p* < 0.001). Elapsed time (β = 0.13; *p* = 0.048), financial strain (β = 0.18; *p* = 0.007), quality of employee-employer communication (β = -0.22; *p* = 0.002), work-centrality (β = 0.33; *p* < 0.001), and organizational commitment (β = 0.16; *p* = 0.04) were all significantly associated with pre-job loss grief reactions. However, work engagement (β = 0.07; *p* = 0.39) and calling (β = 0.05; *p* = 0.60) showed no significant associations with pre-job loss grief reactions in this model. Hence, Hypothesis 5 was partly confirmed.

## Discussion

This study provides valuable insights into the phenomenon of pre-job loss grief reactions. We developed and assessed the validity of a novel instrument, the IJLS, for measuring pre-job loss grief reactions and examined its relation to work attachment.

The results of the scale evaluation of the IJLS provide preliminary evidence that the IJLS has a high reliability and validity for measuring pre-job loss grief reactions, confirming Hypotheses 1 to 4. The findings indicate the IJLS formed a unitary construct, demonstrating both high internal consistency and temporal stability. That pre-job loss grief, depression, and anxiety formed distinct symptom clusters confirmed discriminant validity of the IJLS and aligns with earlier studies on job loss-related grief, depression, and anxiety [7, 8]. The significant associations of pre-job loss grief reactions with depression, anxiety, denial, intolerance of uncertainty, quality of life, acceptance, and optimism supported the scale’s convergent validity, and are consistent with prior studies on job loss-related grief [6, 19]. The association between pre-job loss grief reactions and perceived health was found to be low, indicating that these two variables reflect different constructs among sick-listed employees, which is in line with earlier studies suggesting that job loss and health loss are two separate constructs [9–11].

Regarding work attachment, the study results partially confirmed Hypothesis 5. Work-centrality was positively associated with pre-job loss grief reactions, consistent with earlier findings on job loss-related grief reactions [13]. Although the initial MRA did not show a significant correlation between organizational commitment and pre-job loss grief reactions, the HMRA revealed a significant positive association after controlling for other variables. This suggests that the control variables play an important role in the relationship between work attachment and pre-job loss grief. Specifically, a longer elapsed time in the external job search, increased perceived financial strain, and poorer quality of the employee-employer communication, were associated with higher levels of pre-job loss grief. However, work engagement and calling were not significantly related to pre-job loss grief reactions. This suggests that there may be other factors influencing pre-job loss grief reactions beyond the scope of work attachment.

To gain a deeper understanding of the factors contributing to pre-job loss grief reactions, considering the *psychological contract* between sick-listed employees and their employer could be insightful. This contract involves beliefs and interpretations shaping the mutual agreement, outlining entitlements and fostering feelings of security [47]. Imminent job loss may be perceived as a breach of this contract, where the employer fails to uphold its end [48]. Typically, after one year of absence, sick-listed employees are required to start an external job search, which often coincides with a deterioration in employee-employer communication and a wage reduction to 70%, potentially intensifying financial strain [3]. Consequently, it is conceivable that these circumstances could lead to concerns among sick-listed employees about their future prospects, fostering a sense of urgency, potentially amplifying their emotional distress and organizational commitment [24]. These circumstances may shift the employees’ focus away from work-related aspects, such as work engagement and calling (i.e., finding meaning in work), and towards concerns related to job insecurity, financial strain, and future prospects [49]. This might also explain why there was no effect for work engagement and calling. Moreover, it might be that levels of work engagement and calling deteriorate over time once time away from the job increases. Follow-up studies using a longitudinal design should explore the dynamics of work engagement and calling over time.

Noteworthy is the significant relationship that was found between pre-job loss grief and work status. Participants who worked within their own organization experienced lower levels of grief reactions than participants who worked externally or were not working. No significant difference in intensity of grief reactions was found between participants who worked externally or were not working. These findings could indicate that participants who continue to work internally have a greater sense of hope to remain within their current role or transition to a new position within the organization. Consequently, they experience fewer pre-job loss grief reactions and perceive more control over the outcome of their current situation [50]. Furthermore, these findings suggest that employees’ affiliations with their organizations constitute a part of their identity, and discontinuing affiliations contributes to identity disruption associated with grief.

### Study limitations

Several study limitations warrant consideration. First, the cross-sectional design limits the ability to draw directional conclusions on the relationship between pre-job loss grief reactions and other concepts. However, this limitation did not hinder the objective on developing and validating a novel instrument for measuring pre-job loss grief reactions. Nonetheless, longitudinal methods would be beneficial to better understand the predictive value of the IJLS for work attachment, job loss-related grief, depression, and anxiety symptoms. Additionally, examining pre-job loss grief reactions in relation to the quality of job search behaviour could provide valuable insights, given the previous research emphasis on the significant influence of job search behaviour on successful re-employment [4].

Second, we evaluated aspects of work attachment among sick-listed employees without having a baseline measurement under normal conditions. The current situation may have influenced their perceived level of work-centrality, work engagement, organizational commitment, and meaning of work (i.e., calling). Previous studies indicated that perceived job insecurity was negatively related to work engagement and meaning of work [51], while other research demonstrated that high levels of work commitment combined with a poor organizational climate increased the odds of sickness absence [52]. Exploring the reciprocal relationship between long-term sick leave and work attachment in future studies would provide meaningful perspectives.

Third, we decided to solely incorporate the dedication subscale of the UWES-9 in the present study. Although previous findings indicate strong associations between low scores on the vigour and absorption subscales and subsequently sickness absence [53]. Responding adequately to the vigour and absorption subscales necessitates attendance at work, which is frequently not feasible for sick-listed employees. Nevertheless, in forthcoming studies, it could be valuable to examine the impact of vigour and absorption alongside pre-job loss grief reactions, especially for those who – although partly – remain working in their current job.

Finally, the present sample showed an overrepresentation of highly-educated people (73%), mostly women (84%), compared to the general population in the Netherlands, where long-term sickness absence is highest among people with a primary education [54]. This could have influenced the findings. However, our results showed no significant associations of pre-job loss grief reactions and several socio-demographic factors (except for age), which aligns with earlier results on job loss-related grief reactions [7, 8, 10, 11].

It is important to note that this study employed a convenience sample, warranting caution when generalizing these findings. Since this was the first study to examine the psychometric properties of the IJLS, conducting replication studies would be pertinent for future research.

### Study implications

Despite these limitations, the present study sheds light on the relatively unexplored domain of pre-job loss grief reactions. Our findings indicate that sick-listed employees facing imminent job loss can experience pre-job loss grief reactions. This implies that pre-loss grief reactions, commonly associated with caring for a terminally ill family member [17, 18], can also manifest in cases of imminent job loss. This underscores the importance of recognizing and addressing the emotional impact of impending job loss on sick-listed employees.

Moreover, the IJLS serves as a valuable tool for professionals supporting sick-listed employees in their re-integration process, enabling early detection and necessary support for those experiencing pre-job loss grief. This can also help to increase awareness among professionals (e.g., re-integration advisors, supervisors, and company physicians) as well as sick-listed employees. Importantly, the IJLS has been demonstrated to capture the unique aspects of pre-job loss grief reactions distinct from depression and anxiety, reaffirming its validity and relevance in this context. The IJLS enables scholars to (longitudinally) examine the impact of pre-job loss grief reactions on psychological well-being and job search behaviour, thus enhancing sick-listed employees’ prospects for successful re-employment.

Furthermore, the study bridges the gap between grief literature and the challenges faced by sick-listed employees mandated to seek employment outside their current company. Balancing the uncertainty of returning to their employer, the focus on recovery, and the necessity of starting an external job search may cause severe emotional distress [2, 3]. Our results highlight several potential risk factors associated with pre-job loss grief reactions, including high levels work-centrality and organizational commitment, perceived financial strain, and disrupted employee-employer communication. These factors may become more pronounced as time passes by in the external job search and the end of the sick leave period (104 weeks) draws near, due to the impact of diminishing resources (e.g., time, money, and self-efficacy) [55]. Understanding these dynamics can inform interventions and support systems to better assist sick-listed employees during this challenging period.

## Conclusion

In conclusion, understanding and addressing pre-job loss grief reactions can enhance the re-integration and job prospects of sick-listed employees, improving their psychological well-being and chances of successful re-employment. The development and validation of the IJLS represent a valuable contribution to this endeavour. We advocate for continued exploration of the dynamics of pre-job loss grief reactions and their impact on various outcomes to better support employees during their sick-listed period in future research.

### Supplementary Information

**Supplementary Material 1. **

**Supplementary Material 2. **

## Data Availability

The datasets analysed during the current study are available in the DataverseNL repository, 10.34894/FDDFTJ.

## Abbreviations

CFA: Confirmatory Factor Analysis
CI: Confidence Interval
HADS: Hospital Anxiety and Depression Scale
HMRA: Hierarchical Multiple Regression Analysis
ICC: Intraclass Correlation Coefficient
IJLS: Imminent Job Loss Scale
MRA: Multiple Regression Analysis
RMSEA: Root Mean Square Error of Approximation
SRMR: Standardized Root Mean square Residual
