# Supplementary material for: Pre-job loss grief reactions and work attachment among sick-listed employees: Introduction of the imminent Job Loss Scale
Source: BMC Psychol. 2024 Mar 2;12:118. doi: 10.1186/s40359-024-01626-8 (PMC10909267; doi:10.1186/s40359-024-01626-8)
Supplement: Supplementary file 1 — Supplementary Material 1. [file 40359_2024_1626_MOESM1_ESM.docx]

**Appendix**

The original Dutch version of the Imminent Job Loss Scale is provided here. The English version is presented in Table 2 of the manuscript.

Vragenlijst Dreigend Verlies van Werk

1. Ik verlangde sterk naar hoe mijn leven was voordat ik mijn baan dreigde kwijt te raken

2. Ik dacht voortdurend aan het dreigende verlies van mijn baan

3. Ik was boos over het dreigende verlies van mijn baan

4. Ik kon nauwelijks geloven dat ik mijn baan dreig kwijt te raken

5. Mijn toekomst leek zinloos door het dreigende verlies van mijn baan

6. Ik voelde me emotioneel verdoofd door het dreigende verlies van mijn baan

7. Ik deed er alles aan om maar niet aan het dreigende verlies van mijn baan te hoeven denken

8. Ik wist niet meer goed wie ik was door het dreigende verlies van mijn baan

9. Ik voelde me eenzaam door het dreigende verlies van mijn baan
