# Supplementary material for: Pre-job loss grief reactions and work attachment among sick-listed employees: Introduction of the imminent Job Loss Scale
Source: BMC Psychol. 2024 Mar 2;12:118. doi: 10.1186/s40359-024-01626-8 (PMC10909267; doi:10.1186/s40359-024-01626-8)
Supplement: Supplementary file 2 — Supplementary Material 2. [file 40359_2024_1626_MOESM2_ESM.docx]

**Supplementary File**

Table 1 *Assessment of the imminent job loss scale*

|  | **Comprehensibility (M)** | **Content validity (M)** | **Face validity (M)** | **Total score across  three aspects (M)** |
| --- | --- | --- | --- | --- |
| *Selected items* |  |  |  |  |
| **I longed strongly for how my life was before the impending loss of my job.** | 4.8 | 4.6 | 4.4 | 4.6 |
| **I constantly thought about the impending loss of my job** | 4.8 | 4.8 | 4.5 | 4.7 |
| **I was angry about the impending loss of my job** | 5.0 | 4.8 | 4.6 | 4.6 |
| **I could hardly believe that I am at risk of losing my job** | 4.8 | 4.6 | 4.3 | 4.6 |
| **My future seemed meaningless because of the impending loss of my job** | 4.9 | 4.8 | 4.5 | 4.7 |
| **I felt emotionally numb due to the impending loss of my job** | 4.8 | 4.6 | 4.5 | 4.6 |
| **I did everything I could to avoid thinking about the impending loss of my job** | 4.8 | 4.5 | 4.4 | 4.6 |
| **I no longer knew who I was because of the impending job loss** | 4.8 | 4.7 | 4.4 | 4.6 |
| **I felt lonely because of my impending job loss** | 4.9 | 4.9 | 4.7 | 4.8 |
| *Unselected items* |  |  |  |  |
| The possible loss of my job felt to me like a personal disaster or devastating experience | 4.4 | 4.3 | 4.0 | 4.2 |
| I thought so much about the impending loss of my job that it was difficult for me to do the things I normally do | 4.7 | 4.5 | 4.2 | 4.5 |
| Memories associated with the impending loss of my job upset me | 3.7 | 3.6 | 3.3 | 3.6 |
| I had difficulty accepting the impending loss of my job and its consequences | 4.4 | 4.3 | 3.9 | 4.2 |
| I felt drawn to places and things related to my job | 3.8 | 3.4 | 3.0 | 3.6 |
| I felt baffled by the possible loss of my job | 4.8 | 4.4 | 4.1 | 4.4 |
| Due to the impending loss of my job, I struggled to trust people | 4.6 | 3.9 | 3.7 | 4.1 |
| Due to the impending loss of my job, I felt distance from the people I care about | 4.6 | 3.9 | 3.8 | 4.1 |
| I felt pain in different places in my body due to the possible loss of my job | 4.4 | 3.9 | 3.6 | 4.0 |
| I did everything I could to avoid being reminded of the impending loss of my job and its consequences | 4.4 | 4.4 | 3.8 | 4.2 |
| I found my life empty and meaningless because of the possible loss of my job | 4.7 | 4.7 | 4.1 | 4.5 |
| I was constantly thinking about the impending loss of my job and its consequences | 4.7 | 4.5 | 4.1 | 4.5 |
| I pretended I was not at risk of losing my job | 4.3 | 4.0 | 3.8 | 4.1 |
| I thought it was unfair that others have jobs while I may lose my job | 4.6 | 4.3 | 3.9 | 4.3 |
| I felt bitter about the impending loss of my job and its consequences | 4.4 | 4.2 | 3.9 | 4.2 |
| I envied other people who were not in danger of losing their jobs | 4.6 | 4.1 | 3.9 | 4.2 |
| I felt that my life can only be meaningful if I keep my job. | 4.6 | 4.6 | 4.2 | 4.5 |
| I felt that my image of the world was shattered by the impending loss of my job | 4.2 | 3.7 | 3.5 | 3.8 |
| I had lost my sense of security, confidence or control due to the impending loss of my job | 4.7 | 4.5 | 4.2 | 4.5 |
| The possible loss of my job made me feel tense, irritable or skittish | 4.8 | 4.6 | 4.2 | 4.5 |
| In addition to work, my functioning in other important areas of life had deteriorated due to the impending loss of my job | 4.4 | 4.3 | 3.9 | 4.2 |
| I slept poorly because of the possible loss of my job | 4.8 | 4.6 | 4.2 | 4.5 |
| To avoid thinking about the possible loss of my job, I spent more time behind the PC, watching TV and/or sleeping | 4.3 | 3.8 | 3.7 | 3.9 |
| Memories of the possible loss of my job upset me | 3.9 | 3.9 | 3.6 | 3.8 |
